# To Revise or Not Revise? Isolated Margin Positivity in Localized Pancreatic Ductal Adenocarcinoma

**DOI:** 10.1245/s10434-024-15616-y

**Published:** 2024-06-19

**Authors:** Mohamedraed Elshami, Victoria S. Wu, Henry J. Stitzel, Jonathan J. Hue, Alexander W. Loftus, Ravi K. Kyasaram, John Shanahan, John B. Ammori, Jeffrey M. Hardacre, Lee M. Ocuin

**Affiliations:** 1 Division of Surgical Oncology, Department of Surgery, University Hospitals Cleveland Medical Center, Cleveland, OH USA; 2 Case Western Reserve University School of Medicine, Cleveland, OH USA; 3 Department of Cancer Informatics, University Hospitals Cleveland Medical Center/Seidman Cancer Center, Cleveland, OH USA

**Keywords:** Margin positivity, Surgical margins, Neck margin, Survival benefit, Pancreatic adenocarcinoma

## Abstract

**Background:**

The study determined the proportion of patients with pancreatic adenocarcinoma (PDAC) who had margin-positive disease and no other adverse pathologic findings (APF) using institutional and administrative datasets.

**Methods:**

Patients with clinical stage I or II PDAC in the National Cancer Database (NCDB 2010–2020) and those who underwent pancreatectomy at the authors’ institution (2010–2021) were identified. Isolated margin positivity (IMP) was defined as a positive surgical margin with no APF (negative nodes, no lymphovascular/perineural invasion).

**Results:**

The study included 225 patients from the authors’ institution and 23,598 patients from the NCDB. The margin-positive rates were 21.8% and 20.3%, and the IMP rates were 0.4% and 0.5%, respectively. In the institutional cohort, 68.4% of the patients had recurrence, and most of the patients (65.6%) had distant recurrences. The median recurrence-free survival (RFS) was 63.3 months for no APF, not reached for IMP, 14.8 months for negative margins & 1 APF, 20.3 months for positive margins & 2 APFs, and 12.9 months with all APF positive. The patients in the NCDB with IMP had a lower median OS than the patients with no APF (20.5 vs 390 months), but a higher median OS than those with margin positivity plus 1 APF (20.5 vs 18.0 months) or all those with APF positivity (20.5 vs 15.4 months). Based on institutional rates of IMP, any margin positivity, neck margin positivity (NMP), and no APF, the fraction of patients who might benefit from neck margin revision was 1 in 100,000, and those likely to benefit from any margin revision was 1 in 18,500. In the NCDB, those estimated to derive potential benefit from margin revision was 1 in 25,000.

**Conclusions:**

Isolated margin positivity in resected PDAC is rare, and most patients experience distant recurrence. Revision of IMP appears unlikely to confer benefit to most patients.

**Supplementary Information:**

The online version contains supplementary material available at 10.1245/s10434-024-15616-y.

Pancreatic ductal adenocarcinoma (PDAC) is a highly aggressive cancer with a 5-year overall survival (OS) of 12% among all comers.^1^ The gold standard for surgical treatment of PDAC is a margin-negative (R0) resection, which is crucial for long-term survival for patients who are candidates for surgery.^2–5^ Despite major improvements in surgical management for PDAC and a postoperative mortality rate lower than 5% in specialized centers, patients still encounter a disproportionately high risk of postoperative morbidity and disease recurrence.^6^

Positive microscopic resection margins may be the strongest independent predictor among several prognostic factors associated with long-term survival.^7–10^ The rate of microscopically positive resection margins (R1) is reported to vary from 22.0 to 85.0% in the literature.^11–18^ Several factors can contribute to a positive surgical margin, including definitions based on margin distance, size, and location of the tumor; the presence of lymph node metastasis; and surgical technique.^7,9,10,18,19^

Retrospective studies of intraoperative margin revision in patients with PDAC have reported heterogeneous outcomes. Some studies have demonstrated that additional resection to obtain an R0 margin on permanent section was not associated with improved OS or recurrence-free survival (RFS) for patients undergoing pancreatectomy,^20–24^ whereas others have shown that conversion of intraoperative neck margin-positive cases with revision or even total pancreatectomy may be associated with longer survival.^12,25–27^

Recently, Malleo et al.^28^ reported on 671 patients who underwent pancreatoduodenectomy after neoadjuvant therapy for PDAC with frozen section analysis of neck margin. The authors demonstrated that conversion of an initially positive pancreatic neck margin by additional resection was not associated with oncologic benefits. However, that study included only patients who received neoadjuvant therapy followed by pancreatoduodenectomy and did not quantitatively contribute to the argument concerning the futility of neck margin revision.

In this study, we performed a hybrid analysis using institutional and administrative databases to investigate the frequency of isolated margin positivity (IMP) among patients with localized PDAC who underwent pancreatectomy (pancreatoduodenectomy, distal pancreatectomy, or total pancreatectomy). Our primary aim was to determine the proportion of patients with IMP. Our secondary aims were to determine the fraction of patients who would potentially benefit from revising surgical margins and to examine the association of IMP with OS and RFS.

## Methods

### Institutional Assurances

The Institutional Review Board (IRB) approved this retrospective analysis (STUDY20180966). Data were collected using REDCap, made available by the Clinical and Translational Science Award grant UL1TR002548. Given the retrospective nature of the analysis and minimal harm to the study patients, the need for informed consent was waived. Retrospective analyses of public, deidentified datasets are exempt from IRB review.

### Patient Cohort

We identified patients who underwent a pancreatectomy at our institution between 2010 and 2021. The study enrolled patients with a histologically confirmed diagnosis of PDAC.

In the National Cancer Database (NCDB), patients with PDAC were identified using International Classification of Diseases for Oncology (third edition) histology codes 8140 and 8500. The study included only patients who underwent pancreatectomy (pancreatoduodenectomy, distal pancreatectomy, total pancreatectomy) between 2010 and 2021. The study excluded patients with clinical stage III or IV PDAC at presentation and those with unknown clinical stage. Furthermore, patients were excluded if they had unknown pathologic findings, including Patients with unknown margin status were excluded, nodal positivity, and lymphovascular invasion. Patients also were excluded if they had unknown status for receipt of systemic chemotherapy, radiation therapy, immunotherapy, or palliative care. Finally, patients were excluded if they had unknown sociodemographic characteristics, including race, median income, education, health insurance, facility type, and distance to treating facility. Figure S1 shows the flowchart for selection of the NCDB cohort.

The NCDB contains de-identified patient data and is maintained by the American College of Surgeons and the American Cancer Society. These societies are not responsible for the accuracy of the data presented or the statistical analyses performed. The conclusions reported in this study represent the authors’ interpretation.

### Variables of Interest

The clinicodemographic characteristics included in the institutional dataset were age at surgery, sex, race, clinical stage, pancreatectomy type (pancreatoduodenectomy, distal pancreatectomy, total pancreatectomy), and recurrence patterns (none, local, distant). The treatment-related characteristics included receipt of preoperative therapy, type of preoperative therapy if received (systemic chemotherapy, radiation therapy, or both), chemotherapy regimen if received, and receipt of adjuvant chemotherapy. The pathologic data included surgical margin status, number of positive margins, location of positive margins, lymph node positivity, lymphovascular invasion, and perineural invasion.

The clinicodemographic characteristics in the NCDB were age, sex, race, clinical stage, and pancreatectomy type. The treatment-related characteristics included receipt of systemic chemotherapy (if received, single-agent vs multi-agent and neoadjuvant vs adjuvant chemotherapy) and administration of radiation therapy (if received, neoadjuvant vs adjuvant radiation therapy). The pathologic data included surgical margin status, lymph node positivity, and lymphovascular invasion. The NCDB does not capture perineural invasion or number and location of positive margins.

### Outcome Measures and Definitions

The primary outcome of interest was the proportion of patients with IMP in the institutional and NCDB datasets. Isolated margin positivity was defined as margin positivity (MP) and absence of other adverse pathologic findings, including lymph node metastases, perineural invasion, and lymphovascular invasion (institutional data) or MP and absence of lymph node metastases or lymphovascular invasion (NCDB data). The IMP* in the NCDB was estimated by multiplying IMP by the average rate of no perineural invasion calculated based on the current study as well as other previous studies.^27,29–32^ The average rate of perineural invasion was calculated using the total number of patients with perineural invasion divided by the total number of patients reported in all studies. The secondary outcomes included associations between adverse pathologic findings (all negative, IMP, MP with additional adverse pathologic features, all adverse pathologic features positive), OS, and RFS.

### Clinicopathologic Biostatistical Modeling

In consistency with previous reporting,^33^ a pathologic biostatistical model was devised to estimate the fraction of patients undergoing pancreatectomy who might derive a survival benefit (FDPB) from revising a positive neck margin to negative, thus converting a patient with IMP to having no adverse pathologic findings (NAPF). We estimated FDPB by the following equation:

$${\text{FDPB}} = {\text{IMP}} \times {\text{NMP}} \times {\text{NAPF}},$$

where IMP is the fraction of patients with IMP, NMP is the fraction of patients with neck margin positivity (NMP), and NAPF is the fraction of patients with no adverse pathologic findings.

The FDPB^#^ in the institutional cohort was calculated using MP rather than NMP, where MP is the fraction of patients with positive surgical margins. Therefore, FDPB^#^ = IMP × MP × NAPF.

Similarly, the FDPB* in the NCDB cohort (location of positive margins is not captured) was calculated using MP. Because the NCDB does not also capture perineural invasion, we corrected the rates of IMP and NAPF by multiplying them by the average rate of perineural invasion obtained from the current and previous studies^27,29–32^ to calculate the corrected IMP (i.e., IMP*) and NAPF (i.e., NAPF*). Thus, FDPB* =  MP* × MP × NAPF*.

### Statistical Analysis

Patient characteristics were described as median (interquartile range [IQR]) and compared using the Wilcoxon rank-sum test if they were continuous and non-normally distributed. Categorical variables were described as frequencies and percentages and compared using Pearson’s chi-square test.

In both the institutional and NCDB cohorts, OS was defined as the time from diagnosis to death or last follow-up visit, and RFS was defined as the time from surgery to recurrence in the institutional cohort only because the NCDB does not provide data on RFS. The median OS and RFS were calculated using the Kaplan–Meier method, and comparisons among pathologic categories were performed using the log-rank test.

Multivariable Cox proportional hazards regression analyses were used to examine the association between IMP and OS in the NCDB cohort. The factors included in all multivariable analyses were age, sex, race, education, median income, primary insurance payer, Charlson-Deyo Comorbidity Index, clinical stage, pancreatectomy type, facility type, facility volume (determined based on the total annual hepatopancreatobiliary malignancy case volume, operative and nonoperative, as previously described^34–36^), distance to treating facility, receipt of preoperative therapy, and year of diagnosis. This model was determined a priori based on clinical relevance. All multivariable models were clustered on facility identifiers to account for intra-class correlation for patients nested within the same hospital. Stata 17.0 (StataCorp, College Station, TX, USA) was used for all statistical analyses.

## Results

The study identified 225 patients from our institutional database and 23,598 patients from the NCDB with resected PDAC. In the institutional cohort, 165 patients (73.3%) underwent pancreatoduodenectomy, 43 (19.1%) underwent distal pancreatectomy, and 17 (7.6%) underwent total pancreatectomy. A total of 154 patients (68.4%) had recurrence. Of these recurrences, 34.4% were local and 65.6% were distant. The rate of distant recurrence ranged from 61.1% (*n* = 11/25) of the patients with all pathologic findings positive to 83.3% (*n* = 5/6) of the patients with NAPF (Table 1). As shown in Table S1, 49 patients (21.8%) had positive surgical margins. Of those 49 patients, 37 (74.5%) had one positive margin, and 12 (24.5%) had two positive margins (Table S2). The most common positive margin was the superior mesenteric artery (SMA)/uncinate margin (*n* = 15, 30.6%). Nine patients (18.4%) had a positive neck margin (6 neck margins were analyzed by intraoperative frozen section, and 3 of these margins were positive). None of the three positive margins were revised intraoperatively. Positive lymph nodes were seen in 164 patients (72.9%). Lymphovascular invasion was found in 139 patients (61.8%), and perineural invasion was observed in 190 patients (84.4%).

**Table 1:** Clinicodemographic characteristics of the patients in the institutional cohort

Characteristic	All negative (*n* = 14) *n* (%)	Negative margins with ≥1 adverse pathologic finding (*n* = 162) *n* (%)	IMP (*n* = 1) *n* (%)	Positive margins with ≥1 adverse pathologic finding (*n* = 23) *n* (%)	All positive (*n* = 25) *n* (%)
Median age: years (IQR)	69.5 (60.0–77.0)	67.5 (59.0–75.0)	65.0 (64.0– 66.0)	69.0 (56.0–72.0)	66.0 (58.0–72.0)
Male gender	5 (35.7)	75 (46.3)	0	8 (34.8)	17 (68.0)
White race	13 (92.9)	141 (87.0)	1 (100.0)	18 (78.3)	22 (88.0)
Clinical stage					
I	7 (50.0)	17 (10.5)	0	3 (13.0)	1 (4.0)
II	7 (50.0)	145 (89.5)	1 (100.0)	20 (87.0)	24 (96.0)
Surgical procedure					
Pancreatoduodenectomy	6 (42.9)	125 (77.2)	0	16 (69.6)	18 (82.0)
Distal pancreatectomy	6 (42.9)	29 (17.9)	0	5 (21.7)	3 (12.0)
Total pancreatectomy	2 (14.2)	8 (4.9)	1 (100.0)	2 (8.7)	4 (16.0)
Cancer recurrence	6 (42.9)	117 (72.2)	0	13 (56.5)	18 (72.0)
Recurrence pattern^a^					
Local	1 (16.7)	40 (34.2)	0	5 (38.5)	7 (38.9)
Distant	5 (83.3)	77 (65.8)	0	8 (61.5)	11 (61.1)
Receipt of preoperative therapy	5 (35.7)	38 (23.5)	1 (100.0)	8 (34.8)	4 (16.0)
Type of preoperative therapy					
None	9 (64.3)	124 (76.5)	0	15 (65.2)	21 (84.0)
Chemotherapy	3 (21.4)	34 (21.0)	0	5 (21.7)	4 (16.0)
Chemotherapy and radiation therapy	2 (14.3)	4 (2.5)	1 (100.0)	3 (13.1)	0
Neoadjuvant chemotherapy regimen^b^					
5 FU-based/FOLFIRINOX	2 (40.0)	23 (60.0)	0	5 (62.0)	2 (50.0)
Gemcitabine-based/gemcitabine+nab-paclitaxel	2 (40.0)	14 (37.0)	1 (100.0)	2 (25.0)	2 (50.0)
Both	1 (20.0)	1 (3.0)	0	1 (13.0)	0
Receipt of adjuvant chemotherapy	7 (50.0)	119 (73.5)	0	11 (47.8)	17 (68.0)
Adjuvant chemotherapy regimen^c^					
5 FU-based/FOLFIRINOX	1 (14.3)	12 (10.1)	0	2 (18.2)	3 (17.6)
Gemcitabine-based/gemcitabine+capecitabine	6 (85.7)	104 (87.4)	0	9 (81.8)	13 (76.5)
Both	0	3 (2.5)	0	0	1 (5.9)

IMP, Isolated margin positivity; IQR, Interquartile range; FU, Fluorouracil

^a^The denominator denotes patients who had cancer recurrence

^b^The denominator denotes patients who received neoadjuvant chemotherapy

^c^The denominator denotes patients who received adjuvant chemotherapy

In the NCDB cohort, 16,745 patients (71.0%) underwent pancreatoduodenectomy, 3958 (16.7%) underwent distal pancreatectomy, and 2895 (12.3%) underwent total pancreatectomy (Table 2). The MP rate was 20.3%, and the nodal positivity rate was 64.8% (Table S1). Lymphovascular invasion was found in 49.8%.

**Table 2:** Clinicodemographic characteristics of the patients within the NCDB cohort

Characteristic	All negative (*n* = 5616) *n* (%)	Negative margins with 1 adverse pathologic finding (*n* = 13,184) *n* (%)	IMP (*n* = 744) *n* (%)	Positive margins with 1 adverse pathologic finding (*n* = 1477) *n* (%)	All positive (*n* = 2577) *n* (%)
Median age: years (IQR)	68.0 (61.0–75.0)	67.0 (60.0–74.0)	70.0 (62.0–77.0)	68.0 (60.0–75.0)	68.0 (60.0–74.0)
Male gender	2799 (49.8)	6695 (50.8)	367 (49.3)	764 (51.7)	1389 (53.9)
White race	4538 (80.8)	10,688 (81.1)	573 (77.0)	1187 (80.4)	2085 (80.9)
Clinical stage					
I	3493 (62.2)	6968 (52.9)	416 (55.9)	720 (48.7)	1224 (47.5)
II	2123 (37.8)	6216 (47.1)	328 (44.1)	757 (51.3)	1353 (52.5)
Surgical procedure					
Pancreatoduodenectomy	3616 (64.3)	9530 (72.3)	512 (68.8)	1102 (74.6)	1985 (77.0)
Distal pancreatectomy	1323 (23.6)	2006 (15.2)	166 (22.3)	196 (13.3)	267 (10.4)
Total pancreatectomy	677 (12.1)	1648 (12.5)	66 (8.9)	179 (12.1)	325 (12.6)
Receipt of any systemic chemotherapy	4150 (73.9)	10,304 (78.2)	556 (74.7)	1136 (76.9)	1935 (75.1)
Systemic chemotherapy agent					
None	1466 (26.1)	2880 (21.8)	188 (25.3)	341 (23.1)	642 (24.9)
Single-agent	1896 (33.8)	4771 (36.2)	264 (35.5)	534 (36.2)	908 (35.2)
Multi-agent	2254 (40.1)	5533 (42.0)	292 (39.2)	602 (40.7)	1027 (39.9)
Receipt of neoadjuvant chemotherapy	1573 (37.9)	2163 (21.0)	170 (30.6)	265 (23.3)	299 (15.5)
Receipt of adjuvant chemotherapy	2578 (62.1)	8144 (79.0)	386 (69.4)	872 (76.7)	1635 (84.5)
Receipt of any radiation therapy	1590 (28.3)	3651 (27.7)	335 (45.0)	636 (43.1)	935 (36.3)
Receipt of neoadjuvant radiation therapy	849 (53.4)	779 (21.4)	92 (27.5)	106 (16.7)	59 (6.3)
Receipt of adjuvant radiation therapy	740 (46.6)	2862 (78.6)	242 (72.5)	530 (83.3)	873 (93.7)

NCDB, National Cancer Database; IMP, Isolated margin positivity; IQR, Interquartile range

Only 1 patient (0.4%) in the institutional cohort had IMP compared with 744 patients (3.2%) in the NCDB cohort (Tables 1 and 2). After integration of the average perineural invasion rate (83.7%) into the calculation (Table S3), the IMP* rate in the NCDB cohort was 0.5%. Stratification of the patients by treatment sequence showed that 171 patients (3.8%) in the NCDB cohort who received neoadjuvant therapy had IMP compared with 573 patients (3.0%) who underwent upfront surgery (Table 3). The only patient in the institutional cohort who had IMP received neoadjuvant therapy.

**Table 3:** Summary of patients with different pathologic groups, stratified by treatment sequence

Group	Institutional cohort		NCDB cohort	
	Neoadjuvant therapy (*n* = 56) *n* (%)	Upfront surgery (*n* = 169) *n* (%)	Neoadjuvant therapy (*n* = 5038) *n* (%)	Upfront surgery (*n* = 19,063) *n* (%)
All negative	5 (8.9)	9 (5.3)	1593 (35.1)	4023 (21.1)
Negative margins with at least one adverse pathologic finding	38 (67.9)	124 (73.4)	2201 (48.5)	10,983 (57.7)
Isolated margin positivity	1 (1.8)	0	171 (3.4)	573 (3.0)
Positive margins with at least one adverse pathologic finding	8 (14.3)	15 (8.9)	267 (5.3)	1210 (6.3)
All positive	4 (7.1)	21 (12.4)	303 (6.0)	2274 (11.9)

NCDB, National Cancer Database

**Fig. 1:**
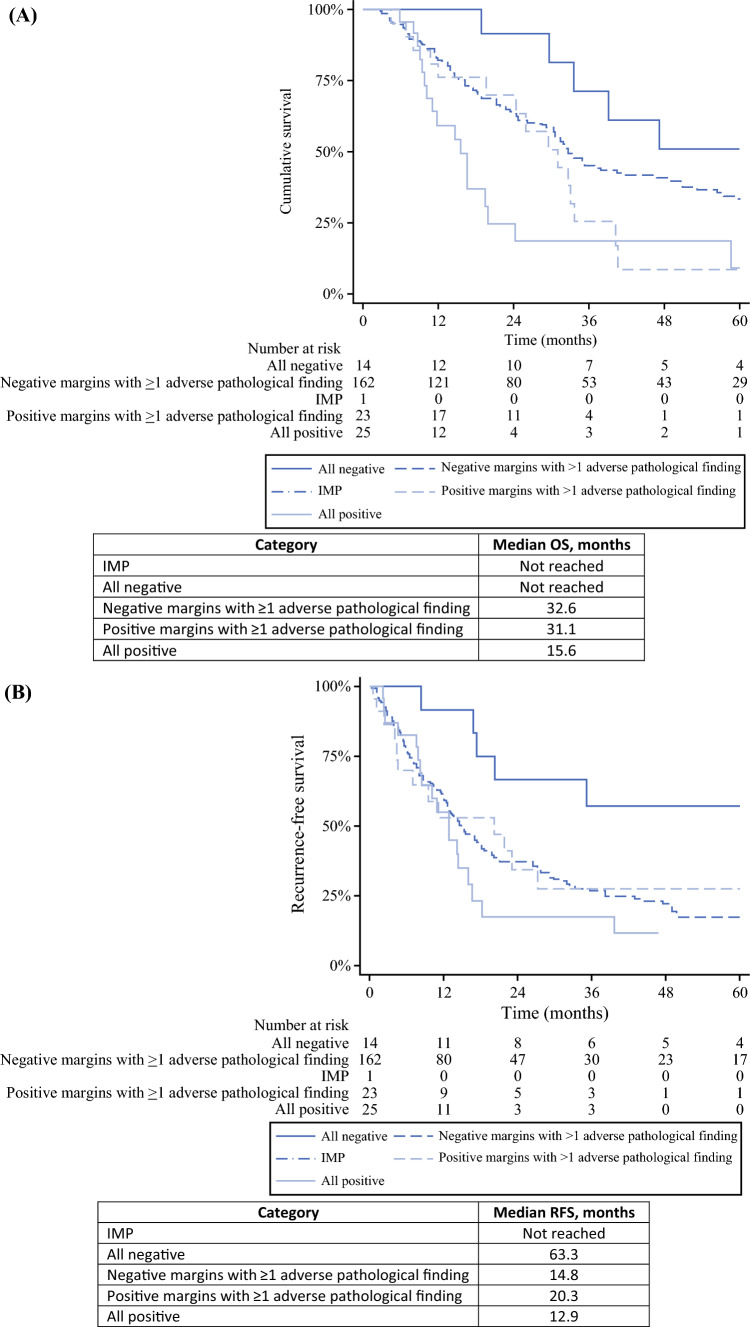

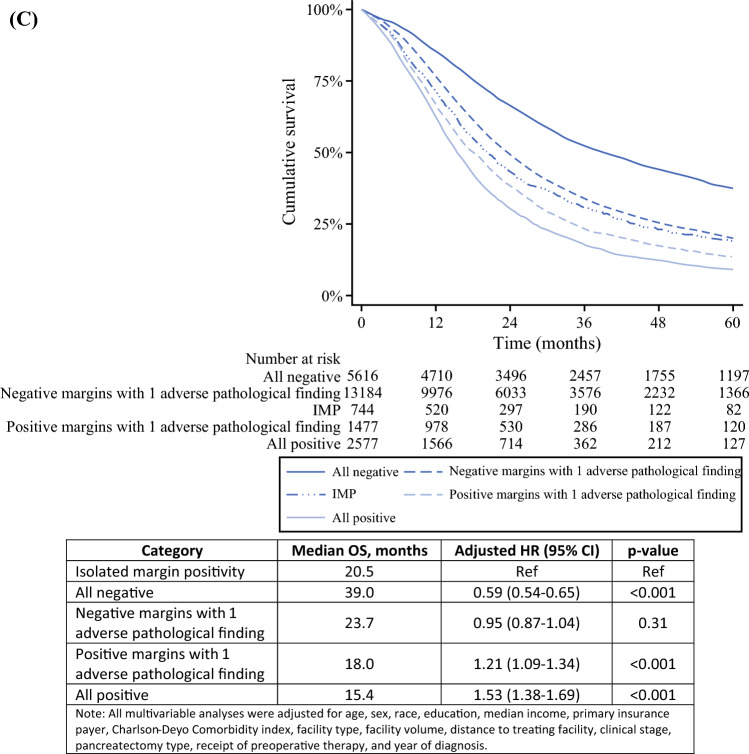
Kaplan–Meier curves estimating (**A**) overall survival in the institutional cohort, stratified by pathologic category, (**B**) recurrence-free survival in the institutional cohort, stratified by pathologic category, and (**C**) overall survival in the National Cancer Database (NCDB) cohort, stratified by pathologic category. *IMP* Isolated margin positivity, *NCDB* National cancer database, *OS* Overall survival, *RFS* Recurrence-free survival, *HR* Hazard ratio, *CI* Confidence interval

In the institutional cohort, the median OS was not reached for IMP or NAPF, but was 32.6 months for negative margins and one adverse pathologic finding, 31.1 months for MP and one or more adverse pathologic findings, and 15.6 months for all positive pathologic findings (Fig. 1A). The median RFS was not reached for IMP, 63.3 months for NAPF, 14.8 months for negative margins and one adverse pathologic finding, 20.3 months for MP and one or more adverse pathologic findings, and 12.9 months for all positive pathologic findings (Fig. 1B).

The patients the NCDB cohort with IMP had shorter OS than the patients with NAPF (median OS, 20.5 vs 39.0 months; hazard ratio [HR], 0.59; 95% confidence interval [CI],: 0.54–0.65; Fig. 1C), but a longer OS than the patients with MP and positive lymph nodes or lymphovascular invasion (median OS, 20.5 vs 18.0 months; HR, 1.21; 95% CI, 1.09–1.34) or those with all positive pathologic findings (median OS, 20.5 vs 15.4 months; HR, 1.53; 95% CI, 1.38–1.69).

We then used our biostatistical models to estimate the fraction of patients who might derive benefit from margin revision. In the institutional cohort, the FDPB was calculated using the following values: IMP, 0.4%; NMP, 4.0%; and NAPF, 6.2%. Thus, FDPB was 0.4% × 4.0% × 6.2%. Therefore, 1 in 100,000 patients might derive a potential survival benefit from revision of a positive pancreatic neck margin (Table 4A). If MP (rather than NMP) were to be included in the equation, FDPB^#^ would equal 0.4% × 21.8% × 6.2%. Therefore, approximately 1 in 18,500 patients might benefit from revision of any positive margin. Among the patients who received neoadjuvant therapy, the fraction of patients who potentially would benefit from revision of a positive neck margin was 1.8% × 7.1% × 8.9%, which is approximately 1 in 9000 patients.

**Table 4:** Summary of the calculated number of patients who would potentially benefit from revising surgical margins

Group	Rate of IMP (%)	Rate of NMP (%)	Rate of MP (%)	Rate of NAPF (%)	FDPB	FDPB^#^
(A) Institutional cohort						
All patients	0.4	4.0	21.8	6.2	1/100,000	1/18,519
Patients with preoperative therapy	1.8	7.1	23.2	8.9	1/9091	1/2703

Group	Rate of IMP (%)	Rate of IMP* (%)	Rate of MP (%)	Rate of NAPF (%)	Rate of NAPF* (%)	FDPB*
(B) NCDB cohort						
All NCDB patients	3.2	0.5	20.3	23.8	3.9	1/25,000
NCDB patients with preoperative therapy	3.8	0.6	16.3	35.1	5.7	1/17,857
NCDB patients with upfront surgery	3.0	0.5	21.3	21.0	3.4	1/27,778

IMP, Isolated margin positivity; NMP, Neck margin positivity; MP, Margin positivity; NAPF, Rate of no adverse pathologic findings; IMP*, Isolated margin positivity rate multiplied by average rate of perineural invasion; NAPF*, No adverse pathologic findings multiplied by average rate of perineural invasion; FDPB, Fraction of patients who might derive a potential benefit after revising neck margin calculated as FDPB = IMP × NMP × NAPF; FDPB^#^, Fraction of patients who may derive a potential benefit after revising all surgical margins calculated as FDPB^#^ = IMP × MP × NAPF; FDPB*, Fraction of patients who may derive a potential benefit after revising all surgical margins calculated as FDPB* = IMP* × MP × NAPF*; NCDB, National Cancer Database

In the NCDB cohort, the FDPB* was calculated using the following values: IMP*, 0.5%; MP, 20.3%; and NAPF*, 3.9%. Thus, FDPB* was 0.5% × 20.3% × 3.9%, which is approximately 1 in 25,000 patients (Table 4B), similar to our findings on the institutional level. In addition, the fraction of patients who potentially would benefit from revision of surgical margins was approximately 1 in 18,000 patients receiving neoadjuvant therapy and 1 in 28,000 patients undergoing upfront surgery.

## Discussion

This study demonstrated that IMP is rare, experienced by less than 1% of our institutional cohort and the estimated NCDB cohort. The fractions of patients in the institutional cohort who would potentially benefit from revision of all margins or neck margins were approximately 1 in 18,500 and 1 in 100,000 patients, respectively. Similarly, in the NCDB cohort, the fraction of patients potentially benefiting from revision of surgical margins was 1 in 25,000 patients. Finally, the patients with IMP had an associated decrease in OS versus the patients with NAPF, but had an associated increase in OS versus the patients with MP and positive lymph nodes or lymphovascular invasion or those with all pathologic findings positive.

Prior studies have shown a discrepancy between the results of intraoperative frozen section analysis and the final histologic report.^22,37^ Patients who undergo operative intervention to revise positive resection margins have higher associated complication rates.^37,38^ The pancreatic neck margin is the most straightforward margin to remediate intraoperatively.^39^ However, the optimal management of a microscopically positive pancreatic neck margin on intraoperative frozen-section pathologic examination is a matter of debate. Several studies have reported that margin revision or even total pancreatectomy may be associated with improved survival.^20–24^ Conversely, several other studies have concluded that additional pancreatic resection to achieve an R0 margin does not influence survival outcomes.^12,25–27,40^ Ample existing evidence suggests that margin status is simply a harbinger of disease biology when pancreatectomy is performed by high-volume surgeons.^41–44^ Our study adds quantitatively to this philosophical argument because we demonstrated that IMP is an extremely rare event. Our biostatistical model predicts that 100,000 patients with a positive pancreatic neck margin would need to be revised to potentially benefit one patient. This seems to be impractical.

Although much of the debate centers on the pancreatic neck margin, other margins include the proximal (duodenal/gastric) margin, the distal jejunal margin, the bile duct margin, and the SMA/uncinate margin. These margins are infrequently assessed on intraoperative frozen section and rarely revised. It seems to us that the amount of general attention given to the pancreatic neck margin while ignoring the other potentially revisable margins and non-modifiable pathologic factors is unwarranted. Ghaneh et al.^13^ performed a post hoc analysis of the European Study Group for Pancreatic Cancer (ESPAC)-3 randomized controlled trial data and included 1151 patients with resected PDAC. The authors found that R1 neck and posterior/retroperitoneal and medial/uncinate margins were separately and independently associated with worse OS and RFS. Furthermore, the presence of more than one positive margin was associated with worse OS and RFS, with an increasing number of margin-positive sites associated with progressively worse OS.

The findings of our study must be interpreted with consideration of some limitations. We did not distinguish between R1 less than 1 mm and R1-direct because this information was not available in all pathologic reports. Nonetheless, this classification has previously been accepted by the American Joint Committee on Cancer and is becoming more relevant in terms of patient outcomes.^45^ However, this classification system is not routinely used in intraoperative frozen section analysis.

Another possible limitation was the very small number of patients with IMP in the institutional cohort, which precluded a more formal survival analysis. We attempted to address this by using the NCDB for this purpose. However, the NCDB does not capture perineural invasion or number and site of surgical margins involved.

A further limitation of this study could have been that its definition of IMP was not validated. It would be ideal to validate this definition in an institutional database with recurrence data to identify variables that associate the most with local-only recurrence. We could not perform such validation given the small number of patients with local-only recurrence in our institutional cohort and that the NCDB does not capture local or distant recurrence data.

External validation was beyond the scope of the current study, but we believe our current investigation sets the groundwork for future collaborative multi-institution or consortium-based studies to further this question in greater detail. We also tried to account for the absence of perineural invasion in the NCDB by incorporating the average rate of perineural invasion from the current study and other large published cohorts. However, this may not have been a perfect alternative. Despite these limitations, our findings quantitatively illustrate the rarity of IMP among patients with resected PDAC and the likely futility of intraoperative revision of a positive neck margin.

## Conclusions

Developing IMP in patients with resected PDAC is rare. Patients with IMP were more likely to have a better OS than patients with all pathologic findings positive or at least one adverse pathologic finding but worse OS than patients with no adverse pathologic findings. The fraction of patients who would potentially benefit from revision of positive neck margins is very small, suggesting that routine practice likely is futile.

### Supplementary Information

Below is the link to the electronic supplementary material.

Supplementary file1 (DOCX 81 KB)
