# Supplementary material for: To Revise or Not Revise? Isolated Margin Positivity in Localized Pancreatic Ductal Adenocarcinoma
Source: Ann Surg Oncol. 2024 Jun 19;31(9):6170–9. doi: 10.1245/s10434-024-15616-y (PMC11300499; doi:10.1245/s10434-024-15616-y)
Supplement: Supplementary file 1 — Supplementary file1 (DOCX 81 KB) [file 10434_2024_15616_MOESM1_ESM.docx]

| **Supplementary figure 1:** Flowchart for selecting the NCDB cohort. |
| --- |
| 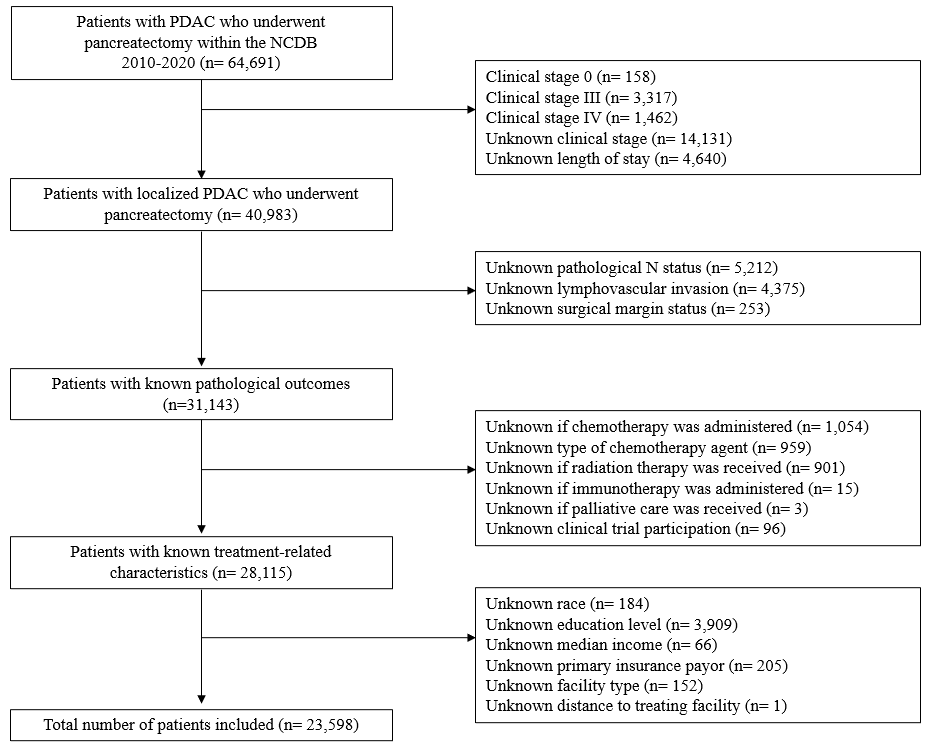 |
| NCDB= national cancer database, n= number of patients. |

| **Supplementary table 1:** Summary of pathological findings. | | |
| --- | --- | --- |
| **Group** | **Institutional cohort**  **(n= 225)** | **NCDB cohort**  **(n= 23,598)** |
| Margin positivity | 49 (21.8) | 4798 (20.3) |
| Nodal positivity | 164 (72.9) | 15295 (64.8) |
| Lymphovascular invasion | 139 (61.8) | 11762 (49.8) |
| Perineural invasion | 190 (84.4) | - |

| **Supplementary table 2:** Summary of number and location of positive margins in the institutional data. | |
| --- | --- |
| **Characteristic** | **n (%)** |
| **Number of positive margins**  1  2 | 37 (75.5)  12 (24.5) |
| **Margin(s) positive**  Uncinate margin  Portal vein groove margin  Posterior retroperitoneal surface of pancreas margin  Neck margin  Anterior margin  Bile duct margin  Portal vein groove and posterior margins  Uncinate and portal vein margins  Proximal and neck margins  Portal vein groove and neck margins  Bile duct and neck margins  Uncinate and neck margins  Bile duct and Portal vein groove margins  Bile duct and posterior margins | 15 (30.6)  8 (16.3)  5 (10.2)  4 (8.2)  4 (8.2)  1 (2.0)  2 (4.1)  3 (6.1)  1 (2.0)  2 (4.1)  1 (2.0)  1 (2.0)  1 (2.0)  1 (2.0) |

| **Supplementary table 3:** Summary of calculating average rate of perineural invasion based on this study as well as previously published cohorts. | | | |
| --- | --- | --- | --- |
| **Study** | **Number of patients included** | **Number of patients with perineural invasion** | **Rate of perineural invasion** |
| Current study | 225 | 190 | 84.4% |
| Ivey et al. | 427 | 234 | 54.8% |
| Groot et al. | 957 | 696 | 89.8% |
| McIntyre et al. | 891 | 814 | 93.0% |
| Zambirinis et al. | 688 | 650 | 94.5% |
| Zhang et al. | 986 | 913 | 92.6% |
| **Average rate of perineural invasion** | 83.8% | | |
